# Galuteolin suppresses proliferation and inflammation in TNF-α-induced RA-FLS cells by activating HMOX1 to regulate IKKβ/NF-κB pathway

**DOI:** 10.1186/s13018-020-02004-x

**Published:** 2020-10-21

**Authors:** Yin Guan, Xiaoqian Zhao, Weiwei Liu, Yue Wang

**Affiliations:** 1 Affiliated Hospital of Nanjing University of Chinese Medicine, Nanjing, 210029 Jiangsu China; 2 Department of Ethics Committee, Affiliated Hospital of Nanjing University of Chinese Medicine, Nanjing, 210029 Jiangsu China; 3 Department of Medical Examination Center, Affiliated Hospital of Nanjing University of Chinese Medicine, Nanjing, 210029 Jiangsu China; 4 Department of Rheumatism Immunity Branch, Affiliated Hospital of Nanjing University of Chinese Medicine, No. 155 Hanzhong Road, Qinhuai, Nanjing, 210029 Jiangsu China

**Keywords:** Rheumatoid arthritis, Galuteolin, Anti-apoptosis, Anti-inflammation

## Abstract

**Objective:**

Galuteolin (Galu) is a substance extracted and purified from honeysuckle. The purpose of this study was to explore the effects of Galu on the TNF-α-induced RA-FLS cells (synoviocytes) and reveal its potential molecular mechanism from the perspectives of anti-apoptosis and anti-inflammation.

**Methods:**

After TNF-α stimulation, cell proliferation of RA-FLS was assessed by CCK-8 assay. TUNEL staining was used to detect the apoptosis. Western blot was used to detect the expressions of Iκκβ, p-p65, p65, p-IκB, IκB, Cleaved-caspase3, Caspase-3, Bcl-2, and Bax. HO-1 were determined by RT-PCR. The contents of pro-inflammatory cytokines IL-1β, IL-6, IL-8, and MMP-1 were determined by ELISA.

**Results:**

Galu significantly suppressed cell proliferation in a dose-dependent manner. Additionally, Galu obviously promotes cell apoptosis rate of RA-FLS cells and elevated the expression levels of HO-1, caspase-3, and Bax, while reducing the expression level of Bcl-2. Furthermore, Galu apparently inhibited the levels of Iκκβ, p-p65, and p-IκB. Moreover, Galu also significantly reduced the levels of pro-inflammatory factors IL-1β, IL-6, IL-8, and MMP-1 in RA-FLS cells.

**Conclusion:**

Galuteolin exerts protective effects against TNF-α-induced RA-FLS cells by inhibiting apoptosis and inflammation, which can guide the clinical use of rheumatoid arthritis.

## Introduction

Rheumatoid arthritis (RA) is a chronic inflammatory joint disease of autoimmune nature, comparing the inflammation of synovium [1]. Macrophage-like synoviocytes and fibroblast-like synoviocytes (FLSs) are crucial ingredients of synovium [2]. Therein, a lot of research about RA has focused on synoviocytes. In this study, tumor necrosis factor (TNF)-α was used to induce the proliferation and inflammatory process in RA-FLS, to explore the therapeutic potential of galuteolin in the treatment of rheumatoid arthritis.

*L. japonica* is an important raw material of *Flos Lonicerae Japonicae* [named Jinyinhua (*Flos Lonicerae*) in Chinese], which has been used as a traditional Chinese medicine for the treatment of rheumatoid arthritis, hepatitis, carbuncles, furuncles, fever, and respiratory infections [3] and contained several active ingredients, such as polyphenols, flavones, and triterpenoid saponins [4]. Galuteolin (Galu, also named luteoloside), belonging to the flavonoids, shows a broad spectrum of biological activities, including anti-bacterial, anti-viral anti-oxidant, anti­inflammatory, anti-nociceptive, and anti-angiogenic effects [5, 6]. Galuteolin is one of two standard compounds officially listed in the Chinese Pharmacopoeia for evaluation of the quality of *L. japonica* [7]. Galuteolin attenuates cerebral ischemia/reperfusion injury in rats via anti-apoptotic, anti-oxidant, and anti-inflammatory mechanisms [8]. Galuteolin exhibited a high activity against influenza virus (H3N2) [9]. Galuteolin could alleviate myocardial cell damage caused by myocardial ischemia-reperfusion by reducing oxidative stress response and inhibiting myocardial cell apoptosis [10]. Galuteolin blocked the activation of NF-κB and TLR2 signaling pathways, thereby reducing the inflammation, damage, and apoptosis of uterine cells caused by staphylococcus aureus [11]. However, the role of Galu in RA has not been widely reported.

Through the inquiry of STITCH website (http://stitch.embl.de, version 5.0), we found that Galu could be combined with protein heme oxygenase 1(HO-1) and regulate HO-1 expression. In the past, knockdown of HO-1 in arthritis rats remarkably eliminated the inhibition of quercetin on inflammatory mediators, including TNF-α, IL-1β, IL-6, PGE2, COX-2, and iNOS [12]. In the HUVECs, HO-1 was related to cell proliferation [13], and HO-1 could increase the invasion of prostate cancer cells in vitro and the growth of tumors in vivo [14]. Therefore, HO-1 is critical for anti-inflammatory and proliferative, which has been confirmed by a research that HO-1 chemical inducer (hemin) significantly reduced oxidative stress and downregulated the expression of pro-inflammatory and pro-fibrogenic genes, including IκκB, NF-κB, monocyte chemotactic protein 1 (MCP-1), and α-smooth muscle actin (α-SMA) [15]. XAN significantly inhibited the proliferation of RA-FLS cells by inhibiting IκκB/NF-κB and downstream target proteins [16]. Silencing miR-136-5p significantly reduced the levels of IL-1, IL-6, TNF-α, IFN-α, IκκB, and NF-κB and ameliorates the inflammatory cell infiltration and damage to the spinal cord [17]. These findings indicated that the IKKβ/NF-κB signal pathway participated in the proliferation and inflammation in RA-FLS cells.

In the present study, we aim to explore the exact protective role of Galu participate in regulating the production of inflammatory factors, cell proliferation, and apoptosis in RA-FLSs. Furthermore, we discuss the roles of Galu on the function of RA-FLS by regulating Iκκβ/NF-κB signal pathway.

## Materials and methods

### Cell culture and treatments

RA-FLS (BNCC340230), rheumatoid arthritis fibroblast synovial cells, were purchased from BeNa Culture Collection (BNCC, Beijing, China) and grown in DMEM medium supplemented with 10% FBS under ordinary conditions at 37 °C in a humidified atmosphere with 5% CO_2_. Cells with 5–10th passages were used.

Galuteolin (Galu) with purity of 99.7% was purchased from Chengdu Herbpurify Co., Ltd. (Chengdu, China). RA-FLS cells were predisposed with Gal at various concentrations (20, 50, 100, and 200 μM) as prearrangements for 30 min and then treated with TNF-α (50 ng/mL) for an additional 1 h.

### Cell transfections

RA-FLS cells were transfected with two different HO-1 siRNA recombinant vectors (si-HO-1-1 and si-HO-1-2) (GenePharma, China) to knockdown HO-1 and empty plasmid as a negative control (si-NC) using Lipofectamine 3000 (Thermo Fisher Scientific). The interference efficiency of these two plasmids was detected by RT-PCR to select a plasmid with the best transfection effect.

### Cell viability assay

RA-FLS cells (1 × 10^4^ cells/well) were seeded in a 96-wells culture plate of 100 μL of culture medium and maintained overnight for attachment. After different treatments, cells were washed with PBS and then treated with 10 μL of Cell Counting Kit-8 solution (Dojindo Laboratories, Japan) at 37 °C for 2 h. The optical density (OD) value of samples was determined by a microplate spectrophotometer (BIO-RAD Science Co. Ltd., USA) at 450 nm. Cell viability was expressed as the percentage of absorbance compared to control cultures.

### Terminal deoxynucleotidyltransferase-mediated nick end labeling (TUNEL) assay

TUNEL staining was performed using “In situ apoptosis detection kit” (Sigma-Aldrich, USA) as per the manufacturer’s protocol. Briefly, RA-FLS cells (2 × 10^4^) were washed with PBS and fixed with 4% paraformaldehyde for 30 min at room temperature. Cells were then incubated for 90 min at 37 °C with terminal deoxynucleotidyltransferase (TdT) incubation buffer. The negative control slide was incubated without the TdT enzyme. The reaction was terminated by washing with PBS, and the slide was examined under fluorescence microscope (Nikon Eclipse 80i, Japan). The experiment was performed three times with three tissue samples.

### Quantitative real-time polymerase chain reaction (qRT-PCR)

After different treatments, total RNA was isolated from RA-FLS cells using TRIzol reagent (Invitrogen, USA). Then, total RNA was further reverse transcribed into cDNA using the Prime Script RT Reagent Kit (Takara, China). The cDNA then served as the template for SYBR Green quantitative real-time polymerase chain reaction (qRT-PCR) (Takara, China) analysis to detect mRNA levels of every gene. The reactions were processed using a 7500 Realtime PCR System (Applied Biosystems, USA) with SYBR Premix Ex Taq Kit (Takara, China). mRNA expression was normalized to the GAPDH level. The specific primers of target mRNA and internal control were designed as following Table 1 [18–20]. Results were shown in form of relative expression calculated by 2^−ΔΔCT^ method. Each experiment was independently performed three times.

**Table 1:** Primer sequences used for qRT-PCR reactions

Gene	Forward primer(5′ to 3′)	Reverse primer(5′ to 3′)
HO-1	5′- AAGACTGCGTTCCTGCTCAAC-3′	5′- AAAGCCCTACAGCAACTGTCG-3′
IL-1β	5′-TTCAGGCAGGCAGTATCACTC-3′	5′-GAAGGTCCACGGGAAAGACAC-3′
IL-6	5′-CCTGAACCTTCCAAAGATGGC-3′	5′-TTCACCAGGCAAGTCTCCTCA-3′
IL-8	5′-ACTGAGAGTGATTGAGAGTGGAC-3′	5′-AACCCTCTGCACCCAGTTTTC-3′
MMP-1	5′-AAAATTACACGCCAGATTTGCC-3′	5′-GGTGTGACATTACTCCAGAGTTG-3′
GAPDH	5′-ATCTCCTTTGTTACCGCTTCC-3′	5′-GAAGATGGTGATGGGATTTC-3′

### Enzyme-linked immunosorbent assay (ELISA)

Levels of IL-1β, IL-6, IL-8, and MMP-1 in RA-FLS cells culture supernatant were measured with commercially available standard sandwich enzyme-linked kits (Beyotime Biotechnology, China) in accordance with the manufacturer’s instructions. Each sample was measured in triplicate.

### Western blot assay

Total protein was extracted from RA-FLS cells using a RIPA kit (Beyotime, China). Harvested cells were lysed on ice. Protein concentrations of the cell supernatants were determined using a BCA Protein Assay kit (Beyotime, China). The samples were then spiked into loading buffer and heated in boiling water for 5 min. Equal amounts of proteins (40 μg) were separated on sodium dodecyl sulfate-polyacrylamide gel electrophoresis polyacrylamide gel electrophoresis (SDS-PAGE) and then transferred to a polyvinylidene fluoride (PVDF) membrane (Millipore, USA). After blocking with 5% non-fat milk in TBST for 2 h at room temperature, membranes were incubated at 4 °C overnight with specific primary antibodies, including anti-Bcl-2 (ab692, Abcam), anti-Bax (ab32503, Abcam), anti-Cleaved-caspase3 (ab49822, Abcam), anti-Caspase-3 (ab13847, Abcam), anti-IKKβ (ab32135, Abcam), anti-p-NF-κB p65 (#3033, Cell Signaling Technology), anti-NF-κB p65 (#3034, Cell Signaling Technology), p-IκB (#2859, Cell Signaling Technology), and IκB (#4812, Cell Signaling Technology). After washed in TBST, membranes were incubated in secondary antibodies (Rabbit or Mouse) at room temperature for 2 h and then were washed again. Specific bands of proteins were visualized using an ECL Plus kit (Millipore, WBKLS0500) with bio-imaging system (Quantity One, version 4.6.2). All experiments were independently performed three times.

### Statistical analysis

All experiments were independently performed three times and data are presented as mean ± standard deviation (SD). Quantile-quantile plots were used to test for normal distribution of data. Statistical analysis was performed using GraphPad Prism 8.0.1 software and performed with one-way ANOVA followed by Tukey’s post hoc test. The value of *P* < 0.05 was considered to be statistically significant.

## Results

### Galuteolin inhibits cell proliferation of RA-FLS cells.

**Fig. 1:**
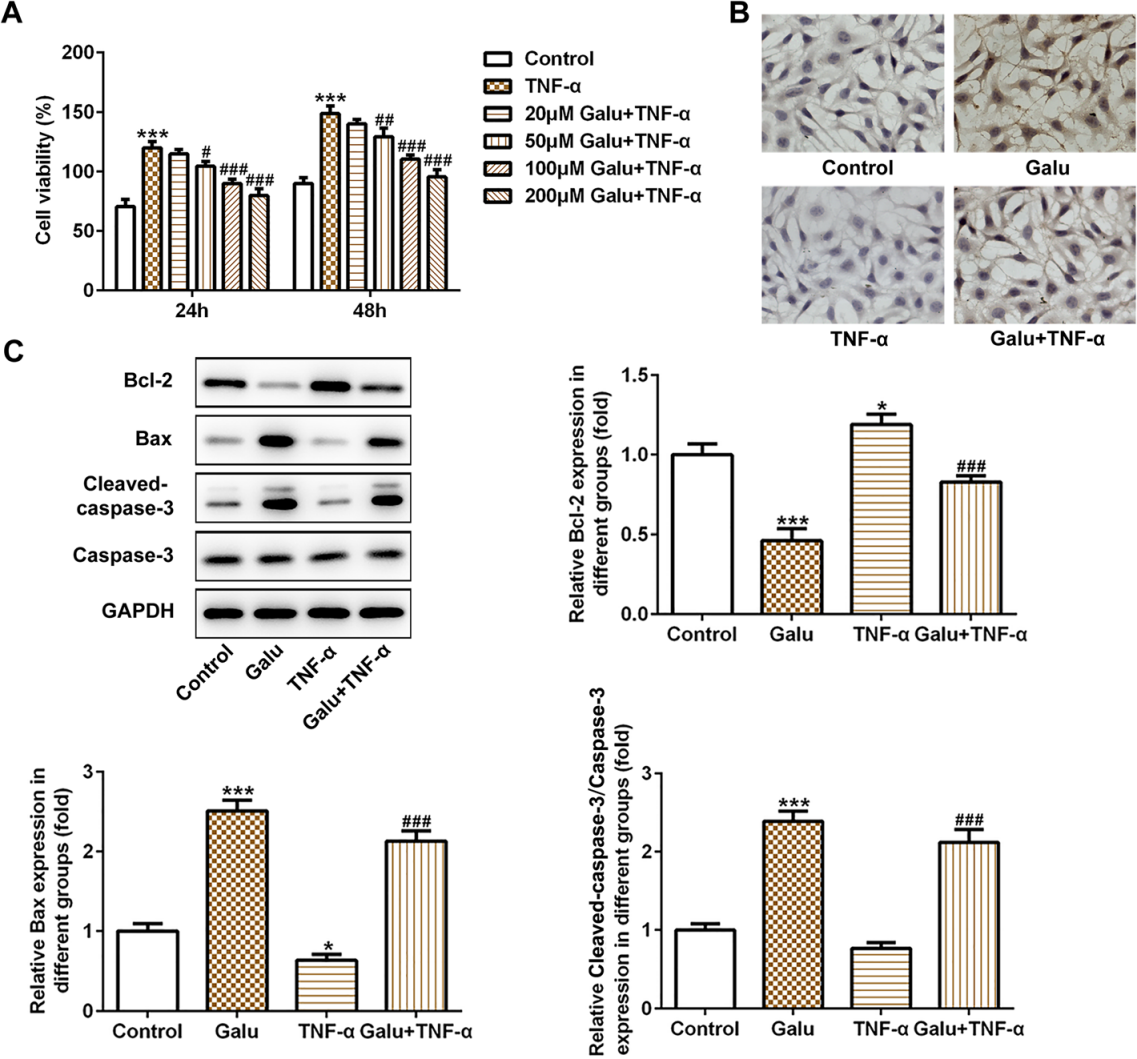
Galuteolin suppressed cell viability and induced cell apoptosis in RA-FLS cells. **a** CCK-8 assay was utilized to measure RA-FLS cell proliferation in TNF-α-induced cells after different concentrations of Galu treatments. **b** TUNEL staining was conducted to detect cell apoptosis in RA-FLS cells in the four groups. **c** Western blot analyses of Bax, Bcl-2 cleaved-caspase-3, and caspase-3 protein expression in RA-FLS cells with indicated treatments. *p < 0.05 and ***p < 0.001 vs. control; ^#^*p* < 0.05, ^##^*p* < 0.01, and ^###^*p* < 0.001 vs. TNF-α

As shown in Fig. 1a, we found that TNF-α (50 ng/mL) induced an obvious increase in the cell viability of RA-FLS cells (*p* < 0.001 at 24 and 48 h). Under the condition of TNF-α treatment, the proliferation of RA-FLS cells was inhibited remarkably by pretreatment of Galu in a concentration-dependent manner (Fig. 1a). These results showed that Galu (20–200 μg/mL) could counteract the auxo-action of TNF-α on the viability of RA-FLS. Therefore, we chose the maximum dose (200 μM) of Galu for the follow-up experiments.

### Galuteolin promotes cell apoptosis of RA-FLS cells.

To verify the effect of Galu on the apoptosis of RA-FLS, TUNEL staining was performed. We found that the ratio of apoptosis (brown represents apoptotic cells) in the Galu-treated alone group was significantly higher than that in the control group, while TNF-α treatment reduced cell apoptosis obviously (Fig. 1b). Interestingly, pretreatment of Galu could counteract the inhibitory role of TNF-α on the apoptosis of RA-FLS cells and induced cell apoptosis (Fig. 1b). These results were further confirmed by detecting the expression of apoptosis-related proteins. As shown in Fig. 1c, Galu could upregulate Bax and cleaved-caspase3 expressions in the RA-FLS cells with TNF-α stimulation or not but suppressed the expression of anti-apoptosis protein Bcl-2. Caspase3 expression levels did not change. These findings demonstrated that galuteolin may have an anti-apoptosis effect in rheumatoid arthritis.

### Galuteolin inhibits inflammation of RA-FLS cells

**Fig. 2:**
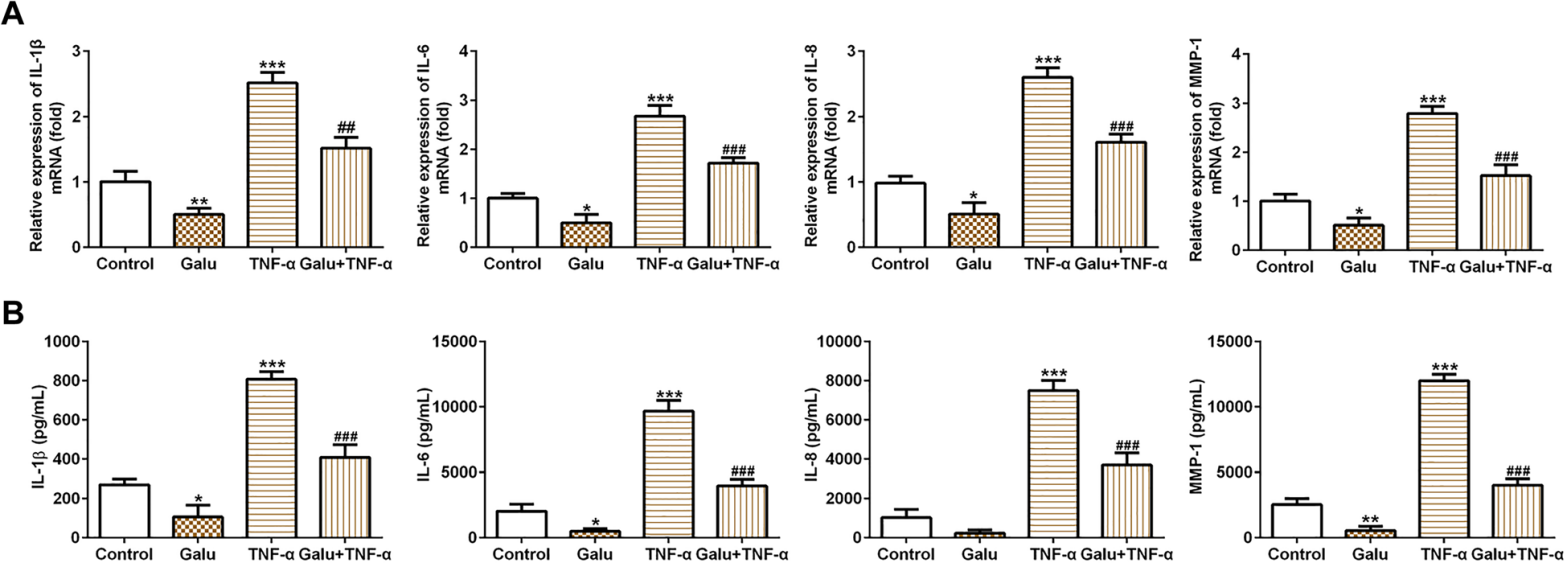
Galuteolin inhibited pro-inflammatory cytokines in RA-FLS cells. **a** The mRNA expression of IL-1β, IL-6, IL-8, and MMP-1 in RA-FLS cells with indicated treatments was measured by RT-qPCR. **b** ELISA was used to test the levels of IL-1β, IL-6, IL-8, and MMP-1 in RA-FLS cells with indicated treatments. *p < 0.05 and ***p < 0.001 vs. control; ^#^*p* < 0.05, ^##^*p* < 0.01, and ^###^*p* < 0.001 vs. TNF-α

Overproductions of pro-inflammatory cytokines have played a key role in the pathophysiology of rheumatoid arthritis. In the present study, we observed that TNF-α could significantly elevate the levels of pro-inflammatory cytokines IL-1β, IL-6, IL-8, and MMP-1. Moreover, Galu suppressed pro-inflammatory cytokine production in the RA-FLS cells induced with or without TNF-α (Fig. 2a, b). Both RT-qPCR and ELISA experiments show this trend, which showed that galuteolin may have a good anti-inflammatory effect for TNF-α-induced inflammation in RA-FLS cells.

### Galuteolin elevated TNF-α-induced inhibition of HO-1 expression in RA-FLS cells

**Fig. 3:**
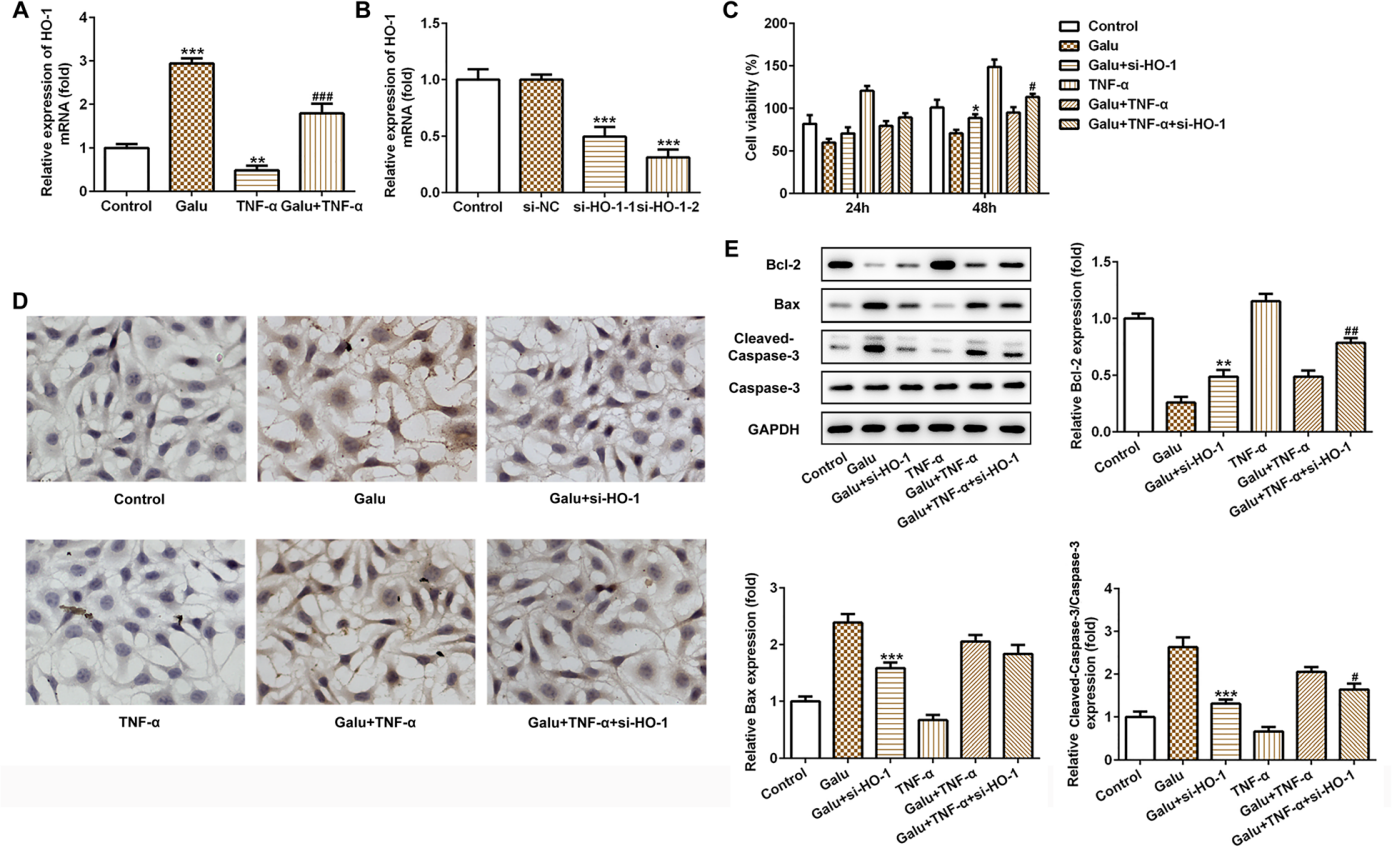
Silence of HO-1 weakened the effects of galuteolin on cell viability and apoptosis of RA-FLS cells. **a** The HO-1 mRNA expression in RA-FLS cells with indicated treatments was measured by RT-qPCR. ***p < 0.001 vs. control; ^###^*p* < 0.001 vs. TNF-α. **b** At 48 h after transfection, RA-FLS cells were collected and the expression of HO-1. **c** Effects of HO-1 siRNA on the proliferation of RA-FLS cells at 24 h, 48 h after Galu pretreatments were evaluated using CCK-8 assay. **d** TUNEL staining assay was performed to investigate the cell apoptosis of RA-FLS cells at 48 h Galu pretreatments. **e** Western blot analyses of Bax, Bcl-2 cleaved-caspase-3, and caspase-3 proteins expression in RA-FLS cells with indicated treatments. *p < 0.05, **p < 0.01, and ***p < 0.001 vs. Galu; ^#^*p* < 0.05 and ^##^*p* < 0.01 vs. Galu + TNF-α

In order to confirm the interaction between Galu and HO-1 and their roles in RA, we examined the expression level of HO-1 in RA-FLS cells after TNF-α stimulation or not. As seen in Fig. 3a, Galu notably upregulated the mRNA expression of HO-1 and TNF-α stimulation suppressed HO-1 expression in RA-FLS cells. Interestingly, the pre-treatment of TNF-α-induced RA-FLS cells with Galu could elevate HO-1 expression when compared with the TNF-α group. These findings confirmed that galuteolin could aggrandize the expression of HO-1 in RA.

### Silencing of HO-1 diminishes the pro-apoptotic effect of galuteolin in RA-FLS cells

To verify whether HO-1 is involved in the pro-apoptotic role of Galu in RA-FLS cells and the relationship between them, the loss-function experiments have proceeded. We first transfected RA-FLS cells with two different small interfering RNAs (si-HO-1-1 and si-HO-1-2) to knocked down HO-1. As shown in Fig. 3b, there was no significant difference between negative plasmid the si-NC group when compared with the control group. However, HO-1 was reduced in si-HO-1-1 and si-HO-1-2 transfected RA-FLS cells when compared with the si-NC group. The plasmid of si-HO-1-2 (si-HO-1) with relatively higher interference efficiency was selected for subsequent experiments.

In addition, knockdown of HO-1 sharply increased cell proliferation, and the inhibitory effect of Galu on cell viability was largely overturned by HO-1 knockdown (Fig. 3c). Besides, TUNEL staining showed that silence of HO-1 could reduce the apoptosis ratio of in Galu-induced cells and could weaken the pro-apoptotic effect of Galu in RA-FLS cells after TNF-α stimulation (Fig. 3d). In the Fig. 3e, using Galu pre-treatment RA-FLS cell group as the control, we found that knockdown of HO-1 remarkably reduced cell apoptosis through downregulating Bax and cleaved-caspase-3 expression and upregulating Bcl-2 expression in the Galu pre-treatment cells. Moreover, after pre-treated with Galu of TNF-α-stimulated RA-FLS cells, the silence of HO-1 could significantly inhibit apoptosis by downregulating Bax and cleaved-caspase-3 expression and increasing Bcl-2 expression, with Caspase-3 unaffected. These findings indicated that HO-1 might be involved in the pro-apoptotic process of galuteolin and as a downstream target of galuteolin in inducing RA-FLS cell apoptosis in response to TNF-α or not.

### Silencing of HO-1 diminishes the anti-inflammatory effect of galuteolin in RA-FLS cells

**Fig. 4:**
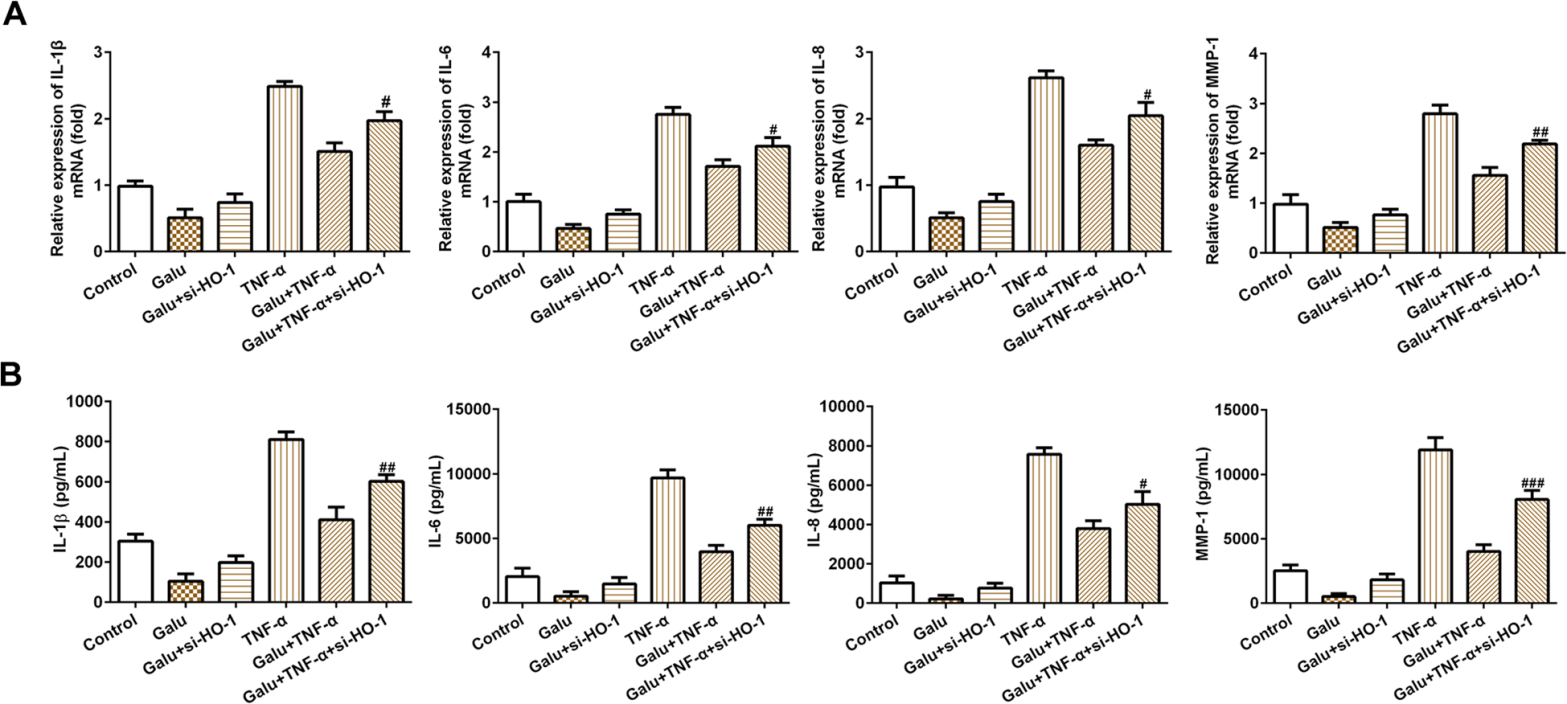
Silence of HO-1 weakened the effects of galuteolin on inflammation response of RA-FLS cells. **a** The mRNA expression of IL-1β, IL-6, IL-8, and MMP-1 was detected after indicated treatments and HO-1 siRNA transfection in RA-FLS cells by RT-qPCR. **b** The levels expression of IL-1β, IL-6, IL-8, and MMP-1 were detected after indicated treatments and HO-1 siRNA transfection in RA-FLS cells by ELISA. *p < 0.05 vs. Galu; ^#^*p* < 0.05 and ^##^*p* < 0.01 and ^###^*p* < 0.001 vs. Galu + TNF-α

As shown in Fig. 4a, the silence of HO-1 sharply increased IL-1β, IL-6, IL-8, and MMP-1 mRNA levels, and the anti-inflammatory effect of Galu was largely overturned by HO-1 knockdown. Similarly, when the expression of HO-1 was suppressed, the effect of Galu to suppress the release of pro-inflammatory cytokines was restored and distinctly enhanced in TNF-α-stimulated cells. In addition, ELISA showed that the levels of IL-1β, IL-6, IL-8, and MMP-1 were upregulated obviously by si-HO-1 plasmid after Galu pretreatment in RA-FLS cells stimulated with or without TNF-α (Fig. 4b). These findings demonstrated that HO-1 might be a downstream target of galuteolin in suppressing inflammation of RA-FLS cells.

### Galuteolin inhibits the Iκκβ/NF-κB signaling pathway via activating the HO-1 expression in RA-FLS cells

**Fig. 5:**
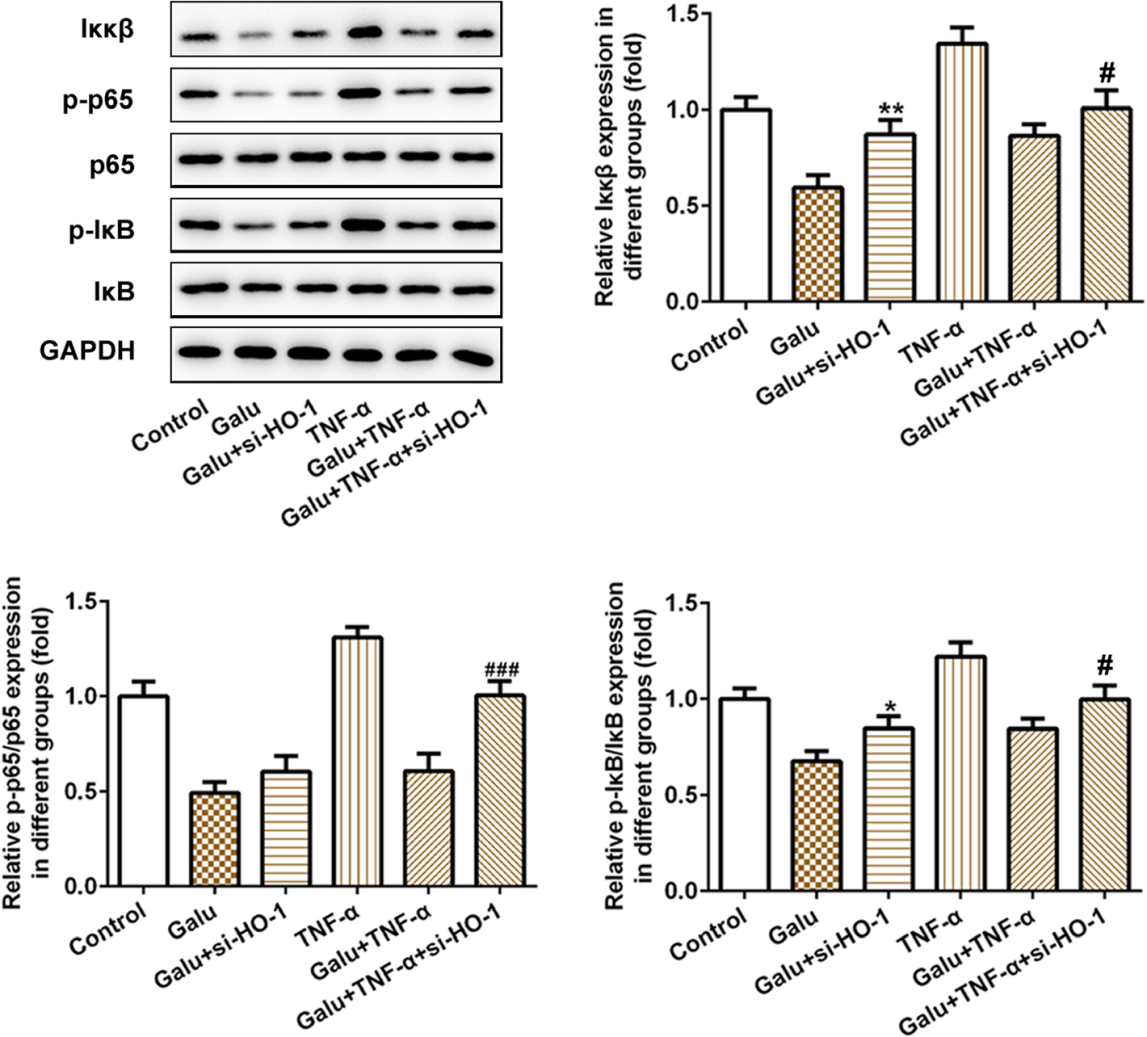
Silence of HO-1 weakened the effects of galuteolin on the IKKβ/NF-κB signaling pathway in RA-FLS cells. Western blot analyses of the IKKβ/NF-κB pathway-related proteins in RA-FLS cells with indicated treatments among six groups. *p < 0.05, **p < 0.01, and ***p < 0.001 vs. Galu; ^#^*p* < 0.05 and ^###^*p* < 0.001 vs. Galu + TNF-α

To explore the Galu targets the downstream signaling pathway behind HO-1, we preliminarily measured the expression level of the Iκκβ/NF-κB signaling pathway. As seen in Fig. 5, we found that Galu could suppress Iκκβ/NF-κB signaling pathway through downregulating Iκκβ, p-NF-κB p65, and p-IκB expression, which were upregulated by si-HO-1 plasmid in the RA-FLS cells. Interestingly, TNF-α led to a sharp increase in the Iκκβ, p-NF-κB p65, and p-IκB expression. Similarly, in the TNF-α-stimulated cells, pre-treatment of Galu could suppress the Iκκβ/NF-κB pathway, which was activated by si-HO-1 transfection.

These findings demonstrated that galuteolin might suppress proliferation and inflammation in TNF-α-induced RA-FLS cells by activating HO-1 to regulate IKKβ/NF-κB pathway.

## Discussion

Rheumatoid arthritis (RA), an autoimmune disorder, was always accompanied by the activation of monocytes/macrophage. Since synovial inflammation is a hallmark of RA, the understanding of synovial biology and pathophysiology may be the best way to approach the pathogenesis of RA which has not yet been fully elucidated [21]. Studies have found that excessive proliferation and inflammatory response and inactive apoptosis can aggravate RA injury, and the main injury mechanism is related to proliferation and apoptosis of synoviocytes, inflammatory reaction, etc. [16, 22]. Despite recent progress in understanding the anti-inflammatory and anti-atherosclerotic effects of galuteolin [8, 23], the molecular mechanism that regulated synoviocytes activities was unknown. In the present study, we comprehensively investigated the role of galuteolin on synoviocytes. Gal-pre-treated RA-FLS cells exhibited a remarkable reduction in cell proliferation and inflammation response and a sharp increase in cell apoptosis. Moreover, galuteolin treatment could upregulate HO-1 expression and suppress the Iκκβ/NF-κB signaling pathway in RA-FLS cells, which suggested the potential of Gal as a treatment for RA.

TNF-α is a powerful proinflammatory cytokine that is overexpressed in the synovial membrane of RA patients. Importantly, anti-TNF-α treatments have introduced the possibility of remission for RA patients [24]. TNF-a, in combination with IL-6, could promote osteoclast differentiation [25]. Herein, we found that TNF-α stimulation caused excessive proliferation and inflammation and deterrent apoptosis in RA-FLS cells, as well as reduced HO-1expression and activated the Iκκβ/NF-κB signaling pathway. Here is the nice thing, all these influences mentioned above caused by TNF-α in RA-FLS cells were attenuated by Gal pre-treatment obviously.

The HO-1, a rate-limiting enzyme for heme degradation, is an important cytoprotective protein. Ample evidence supports the notion that HO-1 can confer protection against oxidative stress and inflammatory and immune responses in joint tissues, which pathway may control the activation and metabolism of articular cells [26]. A previous study showed that salicin ameliorates rheumatoid arthritis by promoting HO-1 expression in RA-FLSs [27]. In our study, a low expression of HO-1 was observed in RA-FLSs and pre-treatment of Gal could elevate HO-1 expression in the presence or absence of TNF-α. Additionally, in the case that the Gal improves the cytopathic state of RA-FLSs, knockdown of HO-1 would reverse the effect of Gal, thus inhibiting cell apoptosis and promoting cell proliferation and inflammatory factor levels. The Iκκβ/NF-κB signaling pathway was expectedly activated by HO-1 silence in RA-FLS cells. This was consistent with previous research that Iκκβ/NF-κB signaling pathway was abnormally activated in RA and played a pathogenic role [16].

The cytokine-induced activation of the NF-κB pathway in FLSs favors survival after ligation of TNF-α receptor. NF-κB helps integrate inflammatory signaling and is important for cell survival in RA. FLS synthesis of MMPs (particularly MMP-1, 3, 8, 13, 14, and 16) promotes disassembly of the type II collagen network, a process that alters glycosaminoglycan content and water retention and leads directly to biomechanical dysfunction. Therefore, galuteolin might suppress proliferation and inflammation through inhibiting the levels of IL-1β, IL-6, IL-8, and MMP-1 in TNF-α-induced RA-FLS cells by activating HO-1 to regulate IKKβ/NF-κB pathway. Similar studies have been widely carried out in RA. Wang et al. found that miR-410-3p upregulation could improve RA through suppressing proliferation, promoting apoptosis, and G1-S phase transition of RA-FLS cells [22]. Tanshinone IIA promoted RA-FLSs apoptosis by enhancing expression of cleaved caspase-3/caspase-9 and inhibiting PI3K/AKT signaling [28]. Andrographolide alleviated murine arthritis by promoting neutrophil apoptosis [29]. Artesunate inhibited chondrocyte proliferation and accelerates cell apoptosis via suppression of the PI3K/AKT/mTOR signaling pathway in rats with RA [30]. Our findings demonstrated the galuteolin treatment accelerated RA-FLS apoptosis through regulating Bax, Bcl-2, and caspase3 expression.

## Conclusion

Our study provides supporting evidence that galuteolin may activate HO-1 and inactivate the IKKβ/NF-κB signaling pathway, resulting in the suppression of synoviocyte proliferation and inflammation as well as the promotion of synoviocyte apoptosis in RA. Our findings assessed the protective effect of galuteolin on RA-FLS cells and its potential molecular mechanism, which provide certain theoretical basis for the treatment of RA with galuteolin. However, due to the limitation of time and funds, we have only explored that galuteolin regulated synoviocyte proliferation, inflammation, and apoptosis of RA-FLS cells via the IKKβ/NF-κB signaling pathway. Further researches are needed to focus on reverse validation test of IKKβ/NF-κB signaling pathway and other signaling pathways to further confirm support the findings in our study.

## Data Availability

The datasets used and/or analyzed during the current study are available from the corresponding author on reasonable request.

## Abbreviations

Galu: Galuteolin
RA: Rheumatoid arthritis
FLS: Fibroblast-like synoviocytes
TNF-α: Tumor necrosis factor α
HO-1: Heme oxygenase 1
